# The Contrasting Role of Extracellular Vesicles in Vascular Inflammation and Tissue Repair

**DOI:** 10.3389/fphar.2019.01479

**Published:** 2019-12-17

**Authors:** Silvia Oggero, Shani Austin-Williams, Lucy Victoria Norling

**Affiliations:** ^1^William Harvey Research Institute, Barts and the London School of Medicine, Queen Mary University of London, London, United Kingdom; ^2^Centre for Inflammation and Therapeutic Innovation Queen Mary University of London, London, United Kingdom

**Keywords:** extracellular vesicles, vascular inflammation, endothelial cell, tissue resolution, tissue repair

## Abstract

Extracellular vesicles are a heterogeneous family of vesicles, generated from different subcellular compartments and released into the extracellular space. Composed of a lipid bilayer encompassing both soluble cytosolic material and nuclear components, these organelles have been recently described as novel regulators of intercellular communication between adjacent and remote cells. Due to their diversified composition and biological content, they portray specific signatures of cellular activation and pathological processes, their potential as diagnostic and prognostic biomarkers has raised significant interest in cardiovascular diseases. Circulating vesicles, especially those released from platelets, leukocytes, and endothelial cells are found to play a critical role in activating several fundamental cells within the vasculature, including endothelial cells and vascular smooth muscle cells. Their intrinsic activity and immunomodulatory properties lends them to not only promote vascular inflammation, but also enhance tissue regeneration, vascular repair, and indeed resolution. In this review we aim to recapitulate the recent findings concerning the roles played by EVs that originate from different circulating cells, with particular reference to their action on the endothelium. We focus herein, on the interaction of platelet and leukocyte EVs with the endothelium. In addition, their potential biological function in promoting tissue resolution and vascular repair will also be discussed.

## Heterogeneity of Extracellular Vesicles

**Figure 1:**
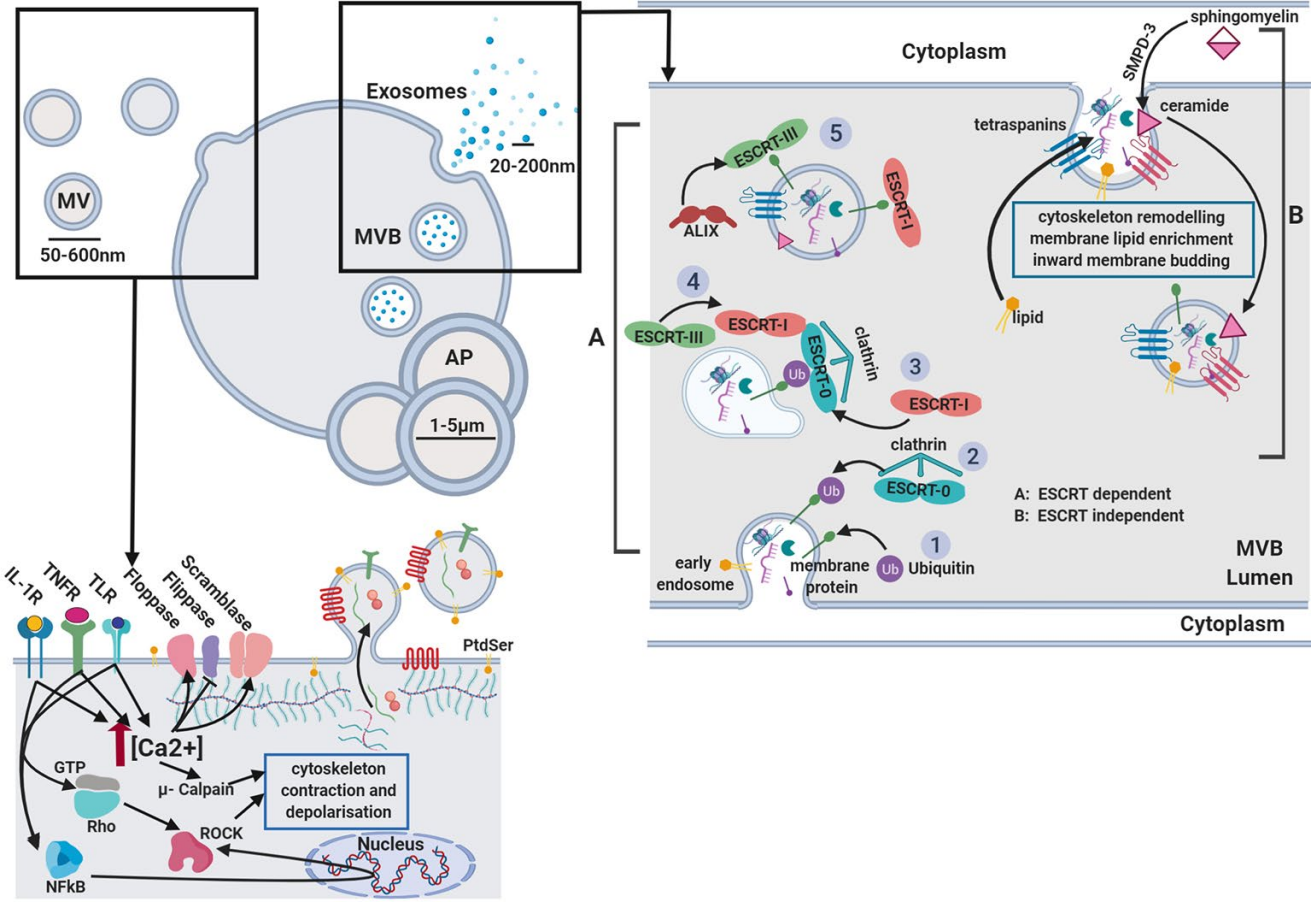
Extracellular vesicle biogenesis. Microvesicles: Activation of receptors coupled to Ca^2+^ signaling promotes phoshatidylserine (PtdSer) exposure on the outer leaflet by modulating flippase, floppase, and scramblase activity. Ca^2+^-activated μ-calpain cleaves cortical actin and activation of Rho-associated protein kinases (ROCK) by RhoA and NFkB induces cytoskeletal contraction. Released vesicles contain membranous proteins, and cytosolic components while budding from the membrane. Exosomes: two separate mechanisms are described. **(A)** ESCRT dependent and **(B)** ESCRT independent biogenesis. GTP, guanine triphosphate; IL-1β, interleukin-1β; PAMP, pathogen-associated molecular pattern; ROCK, Rho-associated protein kinases; TNFα, tumor necrosis factor α; AP, apoptotic body; MVB, multi-vesicular body; TLR, toll like receptor; PtdSer, phosphatidylserine; SMPD-3, sphingomyelin phosphodiesterase 3.

Virtually all cell types have the ability to release small membrane-derived packages of information from their surface (Van der Pol et al., 2012). These nano-packages, termed extracellular vesicles (EVs), represent a key mechanism of paracellular communication (Deatherage and Cookson, 2012) and are attributed numerous roles in regulating both physiological and pathological functions (Van der pol et al., 2012; Yáñez-Mó et al., 2015; Hyenne et al., 2019). First investigated by Peter Wolf in 1967, EVs were described for their prothrombotic functions and coined “platelet dust” (Hargett and Bauer 2013). We now know their functions to be remarkably diverse, largely owing to their generation from distinct processes (Beaudoin and Grondin 1991) resulting in three sub-groups that have overlapping characteristics (Van der Pol et al., 2016). Exosomes being the smallest members of the group, range in size from 40 to 100 nm in diameter. They encompass specific cytosolic products that are packaged into vesicles through the inward budding of multi-vesicular bodies (MVBs) and instead of being degraded, are routed to and released from the plasma membrane (van der Pol et al., 2012). Microvesicles (MV), also termed “microparticles” are larger in size, ranging from 150 to 1,000 nm and are generated through direct budding at the plasma membrane (Van der Pol et al., 2012). The final group member; apoptotic bodies, arise as a consequence of extensive membrane budding that occurs during apoptosis; a homeostatic mechanism typically executed in response to overt-stress or as part of the normal cell life-cycle, with resultant vesicle size ranging from 1 to 5 µm (Elmore, 2007) (**Figure 1**).

EV heterogeneity is derived from their apparent size, membrane composition, and contents; a complex mix of bioactive lipids, RNAs, and proteins (Mack et al., 2000; Ng et al., 2013; Dalli et al., 2013). This diversity is largely dependent on cell activation state and it's environment (Peterson et al., 2008; Dalli et al., 2013). EVs bear markers pertaining to their cell of origin; which can be used to help identify them. EVs not only facilitate the intercellular transfer of cargo, but also membrane-bound receptors and antigen presentation complexes (Li et al., 2013b; Rauschenberger et al., 2016). Intriguingly, EVs can exhibit miRNA, lipid and protein profiles distinct from their cell of origin, suggesting they are not just passive vehicles of the cell’s current proteome, transcriptome, and lipidome, but are actively and specifically packaged with purposeful mediators. Gidlöf *et al.,* revealed active packaging of miR-22 into EVs and its active depletion from platelets with increased activation (Collino et al., 2010; Diehl et al., 2012; Gidlöf et al., 2013). More recent studies have reported single stranded and double stranded DNA in EV fractions (Guescini et al., 2010; Balaj et al., 2011; Thakur et al., 2014). EV-associated DNAs have so far been attributed with the progression of pathology, although this certainly needs more investigation. Additionally, Fonseca et al., described and characterised a variety of metabolic proteins in EV fractions that are able to control the metabolic functions of target cells and tissues (Fonseca et al., 2016), adding another level of complexity to the EV-intercellular signaling paradigm. EVs could therefore be more pertinently considered as discrete extracellular organelles—comprised of a collection of factors that initiate specialised signals in recipient cells (Ludwig and Giebel, 2012; Yáñez-Mó et al., 2015).

Differentiating members of the EV family based on specific characteristics has long been a point of contention in the field. Recently, a systematic and comprehensive proteomic analysis of EVs was performed, using rigorous isolation procedures including flotation in sucrose, iodixanol gradients or immunosorting which has provided a detailed classification system for the different EV subsets (Kowal et al., 2016). This analysis selected large EVs pelleting at low centrifugal speed (2,000×*g*), medium at intermediate speed (10,000×*g*), and small at high speed (100,000×*g*). Among the small EVs, four subcategories were described: EVs coenriched in CD63, CD9, and CD81 alongside endosomal markers; those devoid of CD63 and CD81 but enriched in CD9 (associated with plasma membrane and/or early exosomal markers); those devoid of CD63, CD81, and CD9 and finally those enriched in extracellular proteins or serum proteins. Interestingly, the data would suggest the latter two subtypes do not correlate with markers for exosome biogenesis. Syntenin-1 and TSG101 were associated with tetraspanin-enriched exosomes. In light of these findings, EVs released from different cell types and indeed from the same cells, may be enriched both qualitatively and quantitatively in different tetraspanin content (Kowal et al., 2016). This improved methodology provides a more precise determination of the molecular composition of EVs and suggests that the classical tetraspanin-enriched exosomes may contain a less diverse EV repertoire that previously assumed (Jeppesen et al., 2019).

## The Biogenesis of Extracellular Vesicles

### Exosomes

Exosomes and microvesicles have very distinct methods of biogenesis, although the following description is still up for debate. Two mechanisms of exosome generation have been identified, the endosomal sorting complexes required for transport (ESCRT) dependent and ESCRT independent biogenesis as depicted in **Figure 1**. The ESCRT machinery was first described in dendritic cells (Tamai et al., 2010) for its role in the formation of exosomes and intra-luminal vesicles (ILV). Firstly, ubiquitinated transmembrane cargos are clustered into microdomains of the limiting membrane of MVB by ESCRT-0, ESCRT-I subunits, *via* ESCRT-II, which recruits ESCRT-III sub-complexes to finally enable budding and fusion of this microdomain. The classical ESCRT pathway can interact with syntenin and the ESCRT accessory protein ALIX, which links cargo and the ESCRT-III subunit vacuolar protein sorting-associated protein 32 (VPS32) (Maki et al., 2016). Although the ESCRT-machinery is a well described mechanism for exosome formation, studies show depletion of its components are not sufficient to prevent the production, nor the release of exosomes (Stuffers et al., 2009).

The ceramide-mediated generation of EVs was the first ESCRT-independent mechanism of exosome biogenesis described. Ceramide is negatively charged and impresses a natural negative curvature on the membrane, thus generating membrane subdomains (Goñi and Alonso 2009). Furthermore, ceramide can be metabolised to sphingosine-1-phosphate, activating the G-protein coupled sphingosine-1-phosphate receptor which has been identified as a key player in ILV cargo loading (Kajimoto et al., 2013). Another family of proteins involved in ESCRT-independent exosome biogenesis are the tetraspanins, with particular attention to CD63 which is mainly enriched on the exosome membrane. This process has so far been reported for melanocytes, melanoma cells, and fibroblasts from patients with Down syndrome (Van Niel et al., 2018). Other tetraspanins described to play a role in the formation of microdomains and exosome cargo sorting are: CD81, CD82, and CD9 (Chairoungdua et al., 2010). These proteins can cluster and form dynamic rafts with other cytosolic proteins or other tetraspanins, thus leading to cytoskeletal remodeling and enabling microdomain formation (Buschow et al., 2009; Charrin et al., 2014). However, recent studies underlined how tetraspanins also control the intracellular routing of cargoes, such as integrins in MVBs, which suggests their absence on membranes may also influence exosome generation. Both ESCRT-dependent and independent mechanisms might function in exosome biogenesis and their specific contributions may be different or alter depending on the cell and the cargo (Odintsova et al., 2013).

The involvement of these distinct machineries is also related to the balance between lysosomal degradation and exosome secretion. Indeed, the different components of the ESCRT machinery are related with lysosomal fusion and degradation of MVBs, whilst the syndecan-syntenin-ALIX pathway seems to be restricted to exosome fusion with the plasma membrane and subsequent secretion (Baietti et al., 2012). Recently, calcium dependent SNARE and synaptotagmin family member proteins, have been related with MVB fusion to the plasma membrane in order to release ILVs as exosomes (Hay and Scheller 1997). Of course, there is an indispensable requirement for the cytoskeletal network and the involvement of molecular motors or switches such as myosins, dynein, kinesins, and small GTPases in intracellular transport (Bonifacino and Glick, 2004; Hessvik et al., 2016).

### Plasma Membrane-Derived Extracellular Vesicle Biogenesis

Several pathways are proposed to be involved in the generation of vesicles from the surface of the plasma membrane. Ligand-induced Ca^2+^ influx was one of the first mechanisms described in the exocytosis process. Exogenous stimuli, such as: adenosine triphosphate (ATP), thrombin, and lipopolysaccharide (LPS), all strongly induce intracellular Ca^2+^, triggering potent EV release. This can be significantly reduced with the bivalent cation chelator, BAPTA-AM suggesting any ligand which induces biologically significant Ca^2+^ release into the cytosol could also induce exocytosis. Furthermore, cell surface vesicle release can also be initiated with Ca^2+^ ionophores such as A23187 (Heemskerk et al., 1997; Pasquet et al.,1998).

Plasma membrane rearrangements, including changes in lipid and protein content are also required for the biogenesis of membrane-derived vesicles. Different Ca^2+^ dependent enzymatic machineries such as aminophospholipid translocases (flippase and floppases), scramblases, and calpain are responsible for modifying the membrane phospholipid symmetry and causing physical twisting of the membrane (mostly translocation of phosphatidylserine [PtdSer] from the inner to the outer membrane leaflet), resulting in membrane budding and EV formation (Johnstone et al., 1989) as shown in **Figure 1**. A key scramblase reported for EV budding and PtdSer exposure is TMEM16F, a Ca^2+^-activated chloride channel (Whitlock and Hartzell, 2017). Genetic defects in the activity of this lipid scramblase, suppress the exposure of PtdSer on platelets and thus the production of procoagulant-containing membrane bound vesicles (Suzuki et al., 2010). Despite this, studies have shown vesicle release can still occur even when membrane lipid asymmetry is maintained (Jimenez et al., 2003; Connor et al., 2010). These findings indicate a role for other mechanisms in the formation of membrane-derived EVs. Cholesterol, for example, an important lipid component that regulates membrane fluidity, was observed to be abundant in membrane bound vesicles and depletion impaired vesicle formation in activated neutrophils (Pfrieger and Vitale, 2018).

Of course, in addition to lipids, cytoskeletal components and their regulators are required for membrane bound vesicle biogenesis. The activity of the Rho GTPases and of the Rho-associated protein kinase (ROCK) are required for membrane-derived vesicle biogenesis, due to their important roles as regulators of actin dynamics (Li et al., 2012a); RhoA activation seems to be specifically required during membrane-bound vesicle formation (Ishizaki et al., 1996). RhoA binding to GTP initiates a conformational change in ROCK (Matsui et al., 1996), leading to increased ROCK kinase activity that in turn phosphorylates myosin light chain phosphatase and myosin light chain (Leung et al., 1996). Increased association of myosin light chain to actin filaments results in cytoskeletal contractility and membrane budding (Coleman et al., 2001).

The resultant EVs generated are protected by a phospholipid bilayer, which equips them with a unique protection against rapid degradation, as such they can be utilised at sites close to release or can travel great lengths reaching distant targets. Upon reaching their target, EVs can be taken up by membrane fusion or endocytosis. Hyenne *et al*. demonstrated using a Zebrafish model, that macrophages can extend protrusions to attach and “ferry” tumor-derived EVs towards the cell body before engulfing them (Hyenne et al., 2019). The chosen method of uptake will undoubtedly reflect the markers present on the vesicle surface, although the exact mechanisms for this process remain undescribed.

## Extracellular Vesicles as Biomarkers of Cardiovascular Diseases

EVs are elevated in patients with a number of conditions, including; atherosclerosis (Chironi et al., 2006; Heiss et al., 2008), deep vein thrombosis or pulmonary embolism (Diehl et al., 2001; Rectenwald et al., 2005), cerebrovascular disease (Lee et al., 1993; Jung et al., 2009), or in patients exhibiting several cardiovascular risk factors such as type-2 diabetes mellitus (Koga et al., 2005; Koga et al., 2006), severe hypertension (Preston et al., 2003), and obesity (Goichot et al., 2006). Uncovering the EV parent cell, provides additional and clearer information about the pathophysiology of specific cardiovascular diseases (CVDs). A small case-control study in the PREDIMED trial of patients following a Mediterranean diet observed the concentrations of EVs-derived from different cell types. They found that participants who suffered a cardiovascular event (CVE) within one year of intervention, also presented with elevated EVs derived from lymphocytes and smooth muscle cells (SMC) compared with unaffected participants (Chiva-Blanch et al., 2016). Platelet-derived EVs have also been repeatedly described as good markers for monitoring CVDs. Indeed, the levels of platelet-derived EVs were significantly higher in hypertensive patients, who also have diabetes than in non-diabetic patients. Similarly, a correlation between plasma, platelets, monocytes, or endothelial-derived EVs and hypertensive patients was observed in both the presence and absence of diabetes (Nomura et al., 1995). Another study, showed platelet-derived EVs, but not monocyte-derived EVs, could be considered useful biomarkers for long term follow-up after a myocardial infarction (MI) (Steogonekpień et al., 2012). Increases in endothelial cell (EC)-derived EVs have also been implicated in many diseases with an inflammatory component, such as; atherosclerosis, diabetes, and autoimmune conditions. A direct correlation has been reported between the numbers of endothelial EC-derived EVs and IL-6, indicating a close association with classic inflammatory pathways (Ridger et al., 2017).

The improved ability to purify EVs and acquire accurate information about their numbers lends itself for the identification of new biomarkers, ones that were perhaps previously considered too dilute to be accurately quantified. More recent studies have exploited this to identify miRNAs with prognostic value for CVDs (Zampetaki et al., 2012; Jansen et al., 2014; Kanuri et al., 2018). Patient serum samples from the METEOR trial were tested for the levels of LDL-EVs and their protein content of von Willebrand factor (vWF), serpin C1, and plasminogen X. The METEOR trial sought to determine the effect of rosuvastatin on subclinical atherosclerosis, since patients at risk of CVD with high LDL levels, are often treated with statins. The results from this trial indicated that rosuvastatin-treated patients, have higher levels of plasminogen and vWF in LDL-associated EVs. Serum plasminogen levels were also increased but to a lesser extent, while serum vWF was unaffected (Verbree-Willemsen et al., 2018). Despite the ever-growing literature on EVs, monocyte-derived EVs are poorly discussed within the context of cardiovascular inflammation, especially considering their presence of tissue factor (TF) (Connor et al., 2009; Khaspekova et al., 2016). Patients with meningococcal septic shock, who have suffered from disseminated intravascular sepsis presented with large numbers of monocyte-derived EVs, exposing highly coagulant TF. Furthermore, plasma from sickle cell disease patients, was reported to contain endothelial and monocyte-derived EVs exposing TF, and these EVs were revealed to be procoagulant (Shet et al., 2003). In regards to vascular inflammation, Holvoet *et al*. demonstrated that low levels of mitochondrial cytochrome oxidase, subunit I in circulating CD14^+^ EVs were associated with a higher risk of developing a new CVE in coronary artery disease patients (Holvoet et al., 2016). Also, CD14^+^ EVs were higher in patients with hypertension and with non-ST segment-elevation myocardial infarction (NSTEMI) (Christersson, Thulin, and Siegbahn 2017). In a prospective single-center cohort study, the Athero-Express discovery cohort (1,060 patients), EVs containing cystatin C, serpin G1 and F2, and CD14 were identified as potential biomarkers of secondary CVE (Kanhai et al., 2013). Increased levels of cystatin C, serpin F2, and CD14 were associated with an increased risk of MI, vascular events and all-cause mortality, whereas increased protein content of CD14^+^ EVs also correlated with a higher risk of an ischemic stroke.

## Platelet-Derived Extracellular Vesicles as Biomarkers of Endothelial Dysfunction

Platelet-derived EVs represent the vast majority of EVs in circulation and are thought to originate from both circulating platelets and platelet precursors, which reside in the bone marrow. Although, as we will discuss later, platelet-derived EVs are known to play crucial roles in coagulation, thrombosis, vascular senescence, and permeability, it has also been suggested that circulating platelet-derived EVs can induce vascular dysfunction and by immune modulation, can mediate thrombotic potency of plasma, inducing remodeling of the vasculature. Indeed, elevated levels of platelet-derived EVs were described in patients with acute coronary syndrome, MI, heparin-induced thrombocytopenia, thrombotic complications, and hemolytic uremic syndrome. Whilst abdominal obesity, diabetes mellitus, antiphospholipid syndrome, and sepsis were not associated with increased circulating levels of these EVs (Pinto et al., 2015). In the majority of these studies, it has been proposed that platelet adhesion to leukocytes or endothelial cells, causes activation and subsequent EV release inevitably increasing the inflammatory response (Vagner et al., 2019). In line with this, platelet-derived EVs were proposed to cause dysfunction in arterial hypertension and preeclampsia, although the initial triggers for these pathologies were different; shear stress and sympathetic activity for hypertension and hypoxia/ischemia damage to placental villi for preeclampsia. Regardless of these differences, the result in both pathologies was platelet activation and a subsequent release of vesicles, shown to be the cause of the endothelial dysfunction, vascular remodeling, and increased procoagulative state. Platelet-derived EVs have diagnostic relevance in both hypertension and pregnancy for the prediction of endothelial dysfunction. Platelet-derived EVs were analysed by ELISA in cohorts of pregnant women developing preeclampsia during gestation. The results showed an increase, not only in vWF and endothelin-1, but also in platelet-derived EVs in these women, which suggests EVs represent a valid alternative to already established endothelial biomarkers (Vinayagam et al., 2016). Another study investigating the interconnection between EVs and arterial hypertension, was performed by Preston et al. (2003). Here, they measured the abundance of both endothelial and platelet-derived EVs in patients with untreated severe hypertension and mild hypertension compared to normotensive controls. The concentration of EVs released from endothelial cells and platelets were significantly increased in patients with severe arterial hypertension and this correlated strongly with both systolic and diastolic blood pressure.

Platelet-derived EVs have also been found to predict several complications related to diabetes, another pathology known to be strongly associated with endothelial dysfunction and the occurrence vascular complications, including; vascular endothelial injury and atherosclerosis. Abnormal vascular elasticity and flow-mediated endothelial-dependent dilatation is demonstrated to occur at the early stage of atherosclerosis (Stehouwer et al., 2008). In the context of diabetes, EVs predict the development of cardiovascular pathologies associated with a loss of artery elasticity and the subsequent development of atherosclerotic lesions. As a relevant example, both soluble P-selectin and platelet-derived EV levels were significantly higher in diabetic patients than healthy controls, especially when co-presenting with high levels of LDL (Nomura et al., 1995). The role of LDL in initiating and promoting atherosclerosis has been demonstrated as early as the 90s (Ross, 1986) and Tschope et al., also confirmed that activated platelets play an important role in the development of atherosclerosis in patients with diabetes (Tschoepe et al., 1993). Taken together, these results strengthen the idea that platelet-derived EVs may participate in the development and progression of atherosclerosis in diabetes mellitus, representing a potential candidate biomarker for monitoring these vascular complications. Circulating levels of EVs, including annexinV^+^, platelet, leukocyte, and endothelial-derived EVs were measured in 63 patients with type 2 diabetes and 29 healthy volunteers (Feng et al., 2010). Their findings suggest only endothelial-derived EV levels reflect the endothelial-dependent vascular dilatation and endothelial dysfunction. Platelet-derived EVs positively correlated with postprandial blood glucose levels, indicating that postprandial glucose-dependent platelet activation, occurred in diabetic patients. In line with this, α-glycosidase inhibitor therapy can significantly reduce the circulating levels of platelet-derived EVs (Shimazu et al., 2009). These findings indicate that acute postprandial hyperglycemia in type 2 diabetes may play an important role in platelet activation and that platelet-derived EVs could therefore represent a novel marker for monitoring glucose levels in these patients.

## The Role of Extracellular Vesicles in Endothelial Cell Activation

ECs represent the first barrier of the vessel wall, protecting against pathogen invasion, maintaining vascular integrity and are crucial to vascular homeostasis. Perturbations to the vessel wall including mechanical injury, or systemic factors such as dyslipidemia and smoking can lead EC to alter their phenotype causing vascular remodeling. Endothelial cell homeostasis operates in a tightly controlled balance, with perturbations to this endothelial lining resulting in inappropriate activation and a shift towards a pro-inflammatory state; a major contributor to vascular pathologies.

Platelets are recruited physiologically to injured vessel walls where they become activated and act primarily to prevent blood loss however, their inappropriate recruitment to vessel walls is a contributing factor of many pathologies, including: thrombus formation, hypertension, atherosclerosis, and stroke (Gkaliagkousi et al., 2010; Badimon et al., 2012; Furlan et al., 2016). The role of platelets in modulating endothelial cell function has been increasingly researched over the years and their effects are well described elsewhere (Taraboletti, 1990; Hawrylowicz, 1991; Bustos et al., 2001). It is no wonder then, that platelet-derived EVs also play a pivotal role in endothelial phenotype and function.

### The Action of Platelet-Derived Extracellular Vesicles on the Endothelium

**Figure 2:**
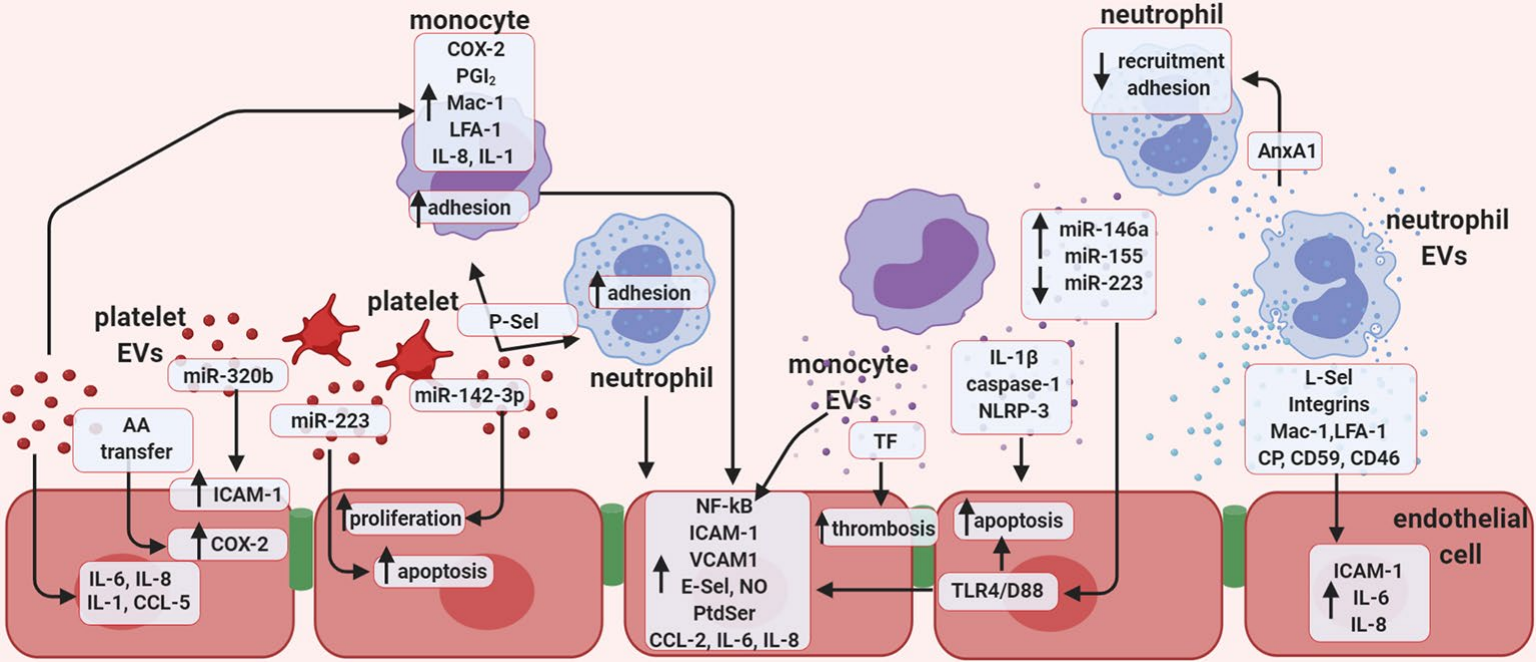
Extracellular vesicles promote endothelial cell activation. Schematic representation of the potential role of *in vitro*-generated extracellular vesicles (EVs), focusing mainly on the role of EVs in vascular inflammation, thrombosis, and regulation of endothelial function. Vesicles of platelet origin can stimulate endothelial cells and leukocytes depending on their cargo of proteins, lipids, and non-coding RNAs. The presence of the microRNAs; miR-223 and miR-142-3p in platelet EVs is important in regulating both proliferation and apoptosis of endothelial cells. In addition, miR-320b can stimulate expression of ICAM-1, while lipid transfer can regulate COX-2 activation. Platelet EVs also increase the release of several cytokines. EVs of platelet origin also promote monocyte inflammation, and together with leukocyte-derived vesicles, favor thrombosis. Monocyte EVs are responsible for activating NF-kB pathway in endothelial cells and enhance expression of selectins, integrins, adhesion molecules, as well as the release of cytokines. Presence of active TF in monocyte EVs also increases thrombosis, while presence of IL-1β, caspase-1, and NLRP-3 induces endothelial apoptosis. EVs released by monocytes promote endothelial inflammation by increasing leukocyte adhesion through the release of several miRNAs. Finally, neutrophil-derived EVs were found to express selectins, integrins, Mac-1, and LFA-1 and are able to enhance endothelial expression of ICAM-1 and release of IL-6 and IL-8. AA, Arachidonic acid; CCL5, C-C motif chemokine 5 (also known as RANTES); COX-2, cyclooxygenase type 2; ICAM-1, intercellular adhesion molecule 1; LFA-1, leukocyte function-associated antigen 1; NO, nitric oxide; PGI_2_; P-Sel, P-selectin; PtdSer, phosphatidylserine; TF, tissue factor; TLR-4/D88, toll like receptor 4; CP, ceruloplasmin.

Platelet-derived EVs are the most abundant type of vesicle found in the circulation under basal conditions, accounting for at least two-thirds of EVs in circulation (Hunter et al., 2008). The ability of platelet-derived EVs to induce increases in surface activation on the endothelium is well-reported, including elevation of the adhesion molecule ICAM-1 (Nomura, 2001; Gidlöf et al., 2013), an effect later ascribed to miR-320b transfer (Gidlöf et al., 2013). Platelet-derived EVs also induce increases in EC cytokine production; IL-8, IL-1, and IL-6, reiterating their enhanced state of activation (Nomura, 2001) (**Figure 2**). Platelet-derived EVs may also facilitate in the destruction of vascular and immune cells in immunoreactive vascular conditions such as immune thrombocytopenia, in which platelets are routinely targeted for removal by the immune system (Cines and McMillan, 2005). Platelet-derived antigens such as HPA-1a can be transferred to endothelial and monocytic cells (THP-1), that readily react with HPA-1a positive antibodies. In this way, these EVs could be responsible for spreading the potential target cell population for autoreactive responses (Majka et al., 2007).

Despite being anucleate, many of the purported effects of platelet-derived EVs can be linked to the delivery of miRNAs. Platelets are now acknowledged to contain approximately 220 different miRNAs (Landry et al., 2009; Kannan et al., 2009) and these can be actively transported to endothelial cells *via* EVs. For example, miR-223 was delivered to EC *via* platelet-derived EVs, resulting in a 22-fold increase in HUVEC miR-223, which was sustained for 48 h (Laffont et al., 2013; Pan et al., 2014). The biological property of EVs to act as intercellular carriers of functional microRNAs has been reiterated by many other groups. Bao *et al*. reported miR-142-3p; which they detected as the most highly expressed microRNA in platelets, could be transferred to EC *via* platelet-derived EVs but not by platelets themselves (Bao et al., 2017). miR-142-3p suppressed the expression of target gene *BCL2L1* and *BCL2*-associated transcription factor (BCLAF1) (Bao et al., 2017; Bao et al., 2018), resulting in increased proliferation and EC apoptosis in an *in vivo* rat model of hypertension. Upregulated EC proliferation and apoptosis play crucial roles in vascular remodeling associated with hypertension (Bao et al., 2017; Bao et al., 2018). In the same vein, the addition of stimulated platelet-derived miR-223 *via* EVs, resulted in a reduction of insulin-like growth factor-1 receptor with subsequent promotion of HUVEC apoptosis, triggered by advanced glycation end products (Pan et al., 2014). This microRNA was found to be elevated in mice with high-fat diet induced atherosclerotic lesions and was elevated in both circulating platelets and platelet-derived EVs in atherosclerosis, hepatitis, enteritis, or nephritis patients (Pan et al., 2014).

Platelets not only contain miRNA, but also the machinery to process pre-miRNA; Dicer, AGO2, and RNA-binding protein 2 (Landry et al., 2009; Laffont et al., 2013), indicating that they are capable of processing pre-miRNA (Warshaw et al., 1967). Unlike leukocyte derived-EVs, platelet-derived EVs possess elevated levels of pre-miRNAs and mature miRNA. Within platelet-derived EVs the concentration of mature miRNA could be enhanced following stimulation with thrombopoietin (Pan et al., 2014). This finding further confirms that platelets are able to convert pre-miRNA into a mature form, which can be actively packaged into vesicles for release. Depending on the method of stimulation, platelets were preferentially depleted of specific miRNAs, leading to the generation of platelet-derived EVs, enhanced in key platelet-miRNAs. Platelets recovered from patient thrombi were depleted of miR-22, much more so than in those recovered from experimentally induced aggregation (Gidlöf et al., 2013). How specific contents are packaged and become enriched in EVs is still unknown, although perhaps activation and therefore upregulated production of these contents is sufficient to cause this seemingly selective packaging.

EVs are an abundant source of lipids, as such, their delivery can regulate lipid metabolism in recipient cells. Platelet-derived EVs are able to initiate lipid metabolism in EC and in platelets through the transport of arachidonic acid (AA). In HUVEC, receiving AA from platelet-derived EVs induced cyclooxygenase-2 (COX-2) activity, resulting in the subsequent generation of prostacyclin (PGI_2_) (Barry et al., 1999), which is known to limit platelet activation and leukocyte recruitment. The initiation of lipid metabolism was also investigated in preeclamptic women. In this case, their EV profiles displayed pro-inflammatory properties; inducing vascular hyporeactivity in vessels of both humans and mice. These effects were also found to be related to interactions between NO and cyclooxygenase systems, leading to endothelial cell activation (Cines and McMillan, 2005).

### Platelet-Derived EVs Modulate Leukocyte Interactions With the Endothelium

Platelets have the ability to interact with other leukocytes in circulation and can affect the way they interact with the endothelium. There are a number of ways by which platelet-derived EVs can alter the adhesiveness of recipient cells and influence their interactions with the endothelium. Firstly, it can be initiated through providing functional P-selectin expressed on the platelet-EV surface, this was demonstrated by Forlow and colleagues (Forlow et al., 2000). Secondary adhesion to vessel walls (leukocytes adhering to other leukocytes already rolling on an endothelial surface), requires interactions mediated through L-selectin. L-selectin interactions induce a rolling state that is faster and more transient than interactions provided by other selectin members (Forlow et al., 2000). The threshold shear stress that is required to optimally support these bonds can be bridged by platelet-derived EVs deposited on the endothelium. These allow maximal tethering of neutrophil-neutrophil interactions by providing P-selectin at a lower shear requirement (Forlow et al., 2000). Given that plasma platelet-derived EV concentrations are elevated in cardiovascular conditions and that physiological concentrations of platelet-derived EVs do not significantly affect endothelial activation, it would suggest that platelet-derived EVs may contribute to increased leukocyte aggregation and adhesion on vessel walls during pathology.

Another mechanism of platelet-derived EV induced adhesiveness occurs by modulating the expression of adhesion molecules. Platelet-derived EVs can upregulate the expression of CD11b and ICAM-1 on THP-1 monocytic cell lines and EC respectively, resulting in increased adhesion between these two cell types. This was observed alongside increases in cytokine transcription and translation from both cells; including IL-8 and IL-1 *β *(Nomura, 2001). In fact, platelet-derived EVs may also directly deliver IL-1 *β* to EC, therefore increasing the adhesiveness of neutrophils to endothelial cells (Lindemann et al., 2001). Other groups have reported the upregulation of adhesion molecules lymphocyte function-associated antigen-1 LFA-1 (CD11a/CD18) and macrophage antigen-1 Mac-1 (CD11b/CD18) on monocytic cells (Barry et al., 1997). One proposed mechanism of platelet derived-EV mediated activation, is through the delivery of AA acting through protein kinase C (PKC) (Barry et al., 1997). A recent paper showed platelet-derived EVs potentiated neutrophil capture under flow, although these interactions were predominantly modulated through P-selectin and integrins (Kuravi et al., 2019). The addition of platelet-derived EVs can also directly recruit immune cells, through the deposition of functional chemokines such as CCL5 (RANTES) to endothelial membranes. CCL5 deposition was found to require a number of receptors on the surface of platelet-derived EVs including sJAM-A and GPIIb/IIIa Fab-fragment, all working in concert (Mause et al., 2005). Increased activation and aggregation of leukocytes on the endothelial vessel wall may potentiate extravasation and inappropriate activation of vessels, contributing to vascular inflammatory disorders. As previously mentioned, lipids play a significant role in modulating the effects of EVs and again, AA from platelet-derived EVs was found to induce eicosanoid formation in monocytic cell lines (U-937) through the induction of COX-2 and corresponded to the increased synthesis of prostaglandin E2 and thromboxane B2. This required PKC signaling, PI 3-kinase, p42 and p44 MAPK, and P38 kinase enzyme abilities (Barry et al., 1997).

### Monocyte-Derived Extracellular Vesicles Activate the Endothelium

Levels of circulating leukocyte-derived EVs are typically low, but do rise precluding an acute or systemic inflammation. The role of activated monocytic-derived EVs on the endothelium was explored by Wang et al. (2011). LPS stimulated THP-1 derived MVs, but not those from unstimulated cells, were able to bind HUVEC and were subsequently internalised, localizing with the EC cytoplasm. This mechanism has previously been suggested to be dependent on CD18a/LFA-1 interactions (Aharon et al., 2008). LPS-induced THP-1-derived MVs, once internalised, induced ERK1/2 and NF-κB signaling pathways as well as the expression of NF-κβ-dependent genes; VCAM-1, ICAM-1 and E-selectin. Active IL-1 *b* could be detected in these LPS stimulated THP-1-derived MVs, but was undetectable in MVs from untreated cells. In accordance with this, higher levels of NLRP3 and caspase-1 were also reported, in this way active inflammasome could initiate inflammatory responses in otherwise resting cells (**Figure 2**). Histological observations indicated that EC incubated with THP-1-derived MVs developed an elongated cellular morphology and gaps appeared between cells after a 20 h incubation. High doses of MVs; 50 µg, resulted in a sharp increase in apoptosis of EC, whist lower doses of 10 µg had little effect (Aharon et al., 2008). Monocyte derived-EV induced EC death has also been described in human pulmonary microvascular endothelial cells (HPMVEC). These EVs contained the active caspase-1 (p20) and induced cell death only when the parent cell was stimulated with LPS, but not under basal conditions. Inhibition of caspase-1 and active caspase-1 was efficient at abrogating these effects (Mitra et al., 2015).

It is pertinent to acknowledge the ability of monocyte-derived EVs to increase thrombogenicity of EC. This occurs initially due to the presence of monocyte-derived EVs increasing exposure of PtdSer on the EC surface. PtdSer acts as a binding site for members of the coagulation pathway, including prothrombinase, TF/VIIa, and tenase. Secondly, increased thrombogenicity occurs through the transfer of TF from monocyte-derived EVs to EC. Interestingly, TF is expressed at higher levels on monocytic-derived EVs, than on monocytes themselves (Aharon et al., 2008). Endothelial TF mRNA expression was also upregulated following coincubation with monocyte-derived EVs—indicating that intervesicular cargo modulated signaling processes to contribute to thrombotic effects exerted by EC (Aharon et al., 2008). Similarly, these EVs also reduced the anticoagulant tissue factor pathway inhibitor and thrombomodulin in EC (Aharon et al., 2008). At an intrinsic level, EVs could therefore play a role in vascular inflammation in hypercoagulable states. Moreover, high glucose was demonstrated to induce EV release from THP-1 cells with functionally intact TF. Mechanistically, both the ERK/MAPK and p38/MAPK pathway were activated after high glucose stimulation, whereas treatment with a p38 inhibitor decreased EV generation by 66% (compared to untreated controls), suggesting a role for p38/MAPK pathway components in regulating the release of monocyte-derived EVs in the context of diabetes (Li et al., 2017). Increased apoptosis of endothelial cells and thrombogenicity, is a hallmark of a number of vascular diseases and is a significant event during atherosclerotic plaque formation (Morel et al., 2006). Monocyte-derived EVs are attributed other interesting properties, such as tube formation when EC were seeded on Matrigel, initiating pro-angiogenic effects (Kim et al., 2004; Aharon et al., 2008).

Intervesicular cargo, specifically miRNA plays a considerable role in the effector functions of membrane bound vesicles. In fact, Dalvi et al., reported monocyte-derived exosomes alter miRNA profile in human brain microvascular endothelial cells (HBMEC). The major constituents of the blood brain barrier; increased miR-146a, miR-146b, miR-155, mir-125a-5p and decreased miR-222 resulting in the induction of inflammatory pathways *via* the TLR4/D88 pathway (Dalvi et al., 2017) (**Figure 2**). It has also been shown that distinct EV miRNA profiles can be generated when monocytes were stimulated with endotoxins and cytokines, which can activate HBMV and upregulate their expression of ICAM-1, VCAM-1, CCL2, and the pro-inflammatory cytokines IL-1β and IL-6, even encouraging monocyte migration. In this way, monocyte-derived EVs may contribute to an inflammatory milieu, disrupting the blood brain barrier and allowing immune cell infiltration; a significant component of many conditions including Alzheimer’s disease, multiple sclerosis, and stroke (Banks et al., 2015).

Pro-inflammatory profiles; ICAM-1, CCL2, and IL-6 expression, can be induced when primary monocyte-derived exosomes are incubated with HUVEC. This effect required NF-κB signaling. Interestingly, IFN-induced monocyte-derived exosomes upregulated the expression of NF-κB but without activation, unlike the generation of exosomes from LPS which showed significant activation (Tang et al., 2016). Cytoplasmic NF-κB could therefore act as a reservoir, allowing its rapid release following additional stimuli, this phenomena has been previously described during macrophage differentiation (Conti et al., 1997). Here we are reminded of the distinct EV profiles that can be generated from a single cellular source. Indeed LPS/Ca^2+^ ionophore-induced monocyte-derived EVs, presented higher expression levels of Tsg101, whilst low endosomal markers were found in EVs obtained after 20 h starvation (Aharon et al., 2008).

In contrast to membrane derived EVs, intraluminal monocyte-derived exosomes were found to maintain their size and generation rate, even in the presence of stimulation. Their contents however, were considerably different (Tang et al., 2016). Monocyte-derived exosomal miRNA content was significantly altered with IFN or LPS stimulation, with significant increases in miR-155, alongside reductions in miR-223 when compared to exosomes generated from non-stimulated cells (Tang et al., 2016) (**Figure 2**). miR-155 has most recently been ascribed pro-inflammatory effects: elevating the expression of pro-inflammatory genes, activating NF-κB, promoting atherosclerosis and foam cell formation (Ma et al., 2011; Bala et al., 2011; Nazari-Jahantigh et al., 2012; Tian et al., 2014). There are conflicting reports on the role of miR-223 in the literature, as previously discussed, platelet-derived miR-223 resulted in reduced insulin-like growth factor-1 receptor and the promotion of apoptosis. It has also been implicated in high-fat diet induced atherosclerotic lesions in *in vivo* models and in patients with atherosclerosis and hepatitis (Pan et al., 2014). However, miR-223 has also been reported to reduce IL-6 and IL-1 β generation in macrophages (Chen et al., 2012), downregulate ICAM-1 (Tabet et al., 2014) and to have anti-inflammatory and cardioprotective effects (Barwari et al., 2016). In this report, decreases in miR-223 alongside increases in miR-155 correlated with increased endothelial cell activation (Tang et al., 2016). It is likely that gene regulation, along with many other exosomal contents and surface molecules, determine the overall fate of cell activation. Despite the conflicting reports on miRNA involvement, it seems exosomes released from monocytes with an activated phenotype could therefore potentiate inflammation under pathological settings. Monocyte-derived EVs have also been implicated in reactive oxygen species (ROS) generation and signaling. Apoptotic THP-1-derived EVs (generated from etoposide incubation for 24 h) were shown to increase nitric oxide (NO) production in endothelial cells in *in vitro* models, although iNOS and superoxide were not affected by EV treatment (Mastronardi et al., 2011). Given that ROS can cause cell damage and dysfunction, the data indicate monocyte-derived EVs can have detrimental effects on endothelial physiology.

### Neutrophil-Derived Extracellular Vesicles Exert Direct Endothelial Actions

Neutrophil-derived EVs appear to share many similarities with monocyte-derived EVs with respect to their effects on the endothelium, although they are less explored than monocyte-derived EVs. Mersi and Altieri in 1998 were the first to investigate this. Neutrophil and EC co-cultures resulted in net increases of the pro-inflammatory cytokines IL-6 and IL-8; a 35-fold increase on the response elicited from each cell type cultured alone (Mesri and Altieri, 1998). Neutrophil-derived EVs were able to indistinguishably replicate the IL-6 response observed in EC, which was unaffected by the presence of a soluble IL-6R inhibitor. Characterisation of neutrophil-derived EVs revealed the expression of a host of surface proteins including selectins, integrins; LFA-1/CD11a, Mac-1/CD11b, and CD62L (L-selectin) as well as regulators of complement; MCP/CD46 and CD59 (Gasser et al., 2003) (**Figure 2**). Neutrophil-derived EVs were found to bind to THP-1 cells and HUVEC in a dose-dependent manner, but were unable to bind erythrocytes, indicating these interactions were receptor mediated.

The majority of studies investigating neutrophil-derived EV actions on EC appear to be centered around activation. Anti-neutrophil cytoplasmic antibodies (ANCAs) are autoantibodies directed against neutrophil antigens and are associated with rare and often fatal conditions called ANCA-associated vasculitis. Elevated levels of EVs are reported in ANCA-associated vasculitis and in patients with active vasculitis (Brogan et al., 2004; Daniel et al., 2006; Erdbruegger et al., 2008; Clarke et al., 2010), specifically CD66b^+^ EVs (Daniel et al., 2006). Hong et al. revealed that stimulating cytokine-primed neutrophils with ANCAs, but not resting neutrophils generated EVs that were phenotypically distinct from those released under basal conditions (Hong et al., 2012). ANCA significantly increased EV generation compared to controls and displayed elevated markers of activation including functionally active CD11b/CD18, myeloperoxidase (MPO) and importantly, ANCA autoantigens. These EVs were able to bind the endothelium, an interaction dependent on β2 integrin CD18 and induced the production of IL-6 and IL-8 in EC. This endothelial activation was not merely dependent on the sheer increase in EV quantity, but also due to distinct content and surface expression as increasing concentrations of resting neutrophil-derived EVs did not affect the endothelium. This research reiterates that the quantity of EVs as a biomarker, may not be sufficient but rather the quality or constituents of these EVs. Similarly, ANCA-stimulated supernatants upregulated endothelial ICAM-1 surface expression due to increased oxidative stress, as indicated by abrogated effects in the presence of antioxidants. Wang et al. revealed independent thrombin generation abilities of neutrophil derived-EVs, which was not exhibited by EVs from resting neutrophils (Wang et al., 2011). ANCA stimulated neutrophil derived-EVs present the potential missing link between how ANCAs can bind EC, despite an EC lacking the respective autoantigens.

### Leukocyte-Derived Extracellular Vesicles Modulate Leukocyte-Endothelial Interactions

Leukocyte-derived EVs are also able to influence other cells in the circulation and affect the way they interact with the endothelium. Our group have previously reported anti-inflammatory and pro-resolutive properties of neutrophil-derived EVs on leukocyte-endothelial interactions. Dalli et al. reported significantly reduced neutrophil recruitment and adhesion to endothelial cell monolayers, when cells were pre-incubated with fMLP generated neutrophil-derived EVs (Dalli et al., 2008) (**Figure 2**). These EVs contained the anti-inflammatory protein Annexin-A1 (AnxA1) and these effects were lost in the presence of a neutralising antibody against AnxA1 or against the AnxA1 receptor; Lipoxin A4 receptor ALX. This anti-inflammatory action was also observed in an *in vivo* air pouch model, where neutrophil recruitment was markedly reduced, again dependent on the action of AnxA1; as WT neutrophil-derived EVs, but not those from AnxA1 null mice were able to elicit a reduction in neutrophil infiltration. In this way, neutrophil-derived EVs act in an autocrine manner to dampen immune activation and promote resolution. This is not the only report of protective neutrophil-derived EVs as they are shown to significantly dampen pro-inflammatory cytokine release from monocytes (Gasser and Schifferli, 2004).

A recent paper revealed divergent effects of exosomes in *in vitro*-exosome mixtures from both HUVEC and monocytic cells generated under high glucose conditions increased ICAM-1 expression on both cell types. However, exosomes generated under basal-conditions from both cell types were able to reverse the high-glucose activation of ICAM-1 (Sáez et al., 2019). Indeed, monocyte-derived EVs increased transient platelet attachment to HUVEC under flow; in part through upregulated vWF expression resulting from the increased generation of ROS; predominantly superoxide (Essayagh et al., 2007). Monocyte/macrophage EVs also accelerate the development of atherosclerosis by decreasing cell migration and promoting macrophage entrapment in the vessel wall *via* delivery of specific miRNAs, e.g. miR-146a (Nguyen et al., 2018).

## Extracellular Vesicles Promote Endothelial Dysfunction At the Cellular and Tissue Level

It is important to highlight how subsets of EVs may influence pathological vascular processes, acting not only on EC, but also on other blood components involved in the development of these pathologies. In line with this, a few studies focusing on atherothrombosis have investigated the role of monocyte-derived EVs and neutrophil-derived EVs in the activation of macrophages; well known to be crucial during plaque formation. Currently, the data regarding monocyte/macrophage-derived EVs collectively suggests a pro-inflammatory role on macrophages, inducing: superoxide anion production, pro-inflammatory cytokine release, NF-κB activation (Bardelli et al., 2012) and apoptosis (Sarkar et al., 2009). Neutrophil-derived EVs were able to block the inflammatory responses of macrophages to zymosan and LPS, due to early-phase TGF-β1 secretion and the surface exposure of PtdSer (Gasser and Schifferli, 2004).

Leukocyte- derived EVs have also been shown to activate SMC. For example, monocyte-derived EVs containing caspase-1 isolated from endotoxin treated cells, were able to induce apoptosis in VSMC. This activity was not derived from pre-treatment with endotoxin, and was inhibited by a caspase-1 inhibitor, but not by inhibitors of Fas-L, IL-1β and IL-18. Moreover, caspase-1 encapsulation was required to guarantee the effect, as disruption of EV structure resulted in the loss of apoptotic activity, but not of caspase-1 enzymatic activity. Thus, monocytes were demonstrated to deliver their cell-death message, dependent upon the release of EVs containing functional caspase-1. Similarly, neutrophil-derived EVs isolated from cells that had been stimulated with LPS could modulate the phenotype of airway SMC in the context of asthma. Briefly, the authors showed the ability of neutrophil-derived EVs to be rapidly internalised by airway SMC and to alter their proliferative capacity, highlighting their potential important role in the progression of asthma and airway remodeling (Vargas et al., 2016). Beside leukocyte-derived EVs platelet-derived EVs were described to be responsible for skewing smooth muscle cells from a contractile, to a synthetic phenotype (Vajen et al., 2017).

EVs also contribute to several vascular pro-inflammatory events at the tissue level. Subsets of EVs are well recognised for their ability to promote coagulation and thrombosis. The production of EVs leads to the surface expression of anionic phospholipids; mainly PtdSer, which enhances their pro-coagulant activity. Indeed, the externalised negatively charged phospholipids contribute to the assembly and the activation of prothrombinase complexes, thus enhancing thrombin formation (Owens and Mackman, 2011). Although PtdSer is exposed on the surface of most EVs, particularly those of platelet origin (Italiano et al., 2010; Flaumenhaft et al., 2010), EVs that stain negatively for annexin V have also been described, demonstrating the existence of PtdSer-negative EV populations. This would indicate the existence of other molecules, expressed by EVs also contribute to their pro-thrombotic role (Perez-Pujol et al., 2007).

The procoagulant activity of different subsets of EVs is further increased by the expression of TF and EVs that bear both PtdSer and TF, have the highest procoagulant activity. TF is a key modulator of the coagulation cascade; its extracellular domain binds and activates factor VII, triggering hemostasis after vascular injury. Interestingly, monocyte-derived EVs are the main source of TF in the blood stream. Several studies have shown the stimulation of monocytes with different pro-inflammatory stimuli, such as angiotensin II or IL-33, enhances EV release and pro-coagulant features (Cordazzo et al., 2013). They also induce a time and concentration-dependent increase in monocyte TF mRNA and protein levels *via* binding to the ST2-receptor and activation of the NF-κB-pathway (Stojkovic et al., 2017). Moreover, the interaction of the endothelium with EVs isolated from monocytes, results in overexpression of TF on the EC surface and a reduction in expression of the anticoagulant tissue factor pathway inhibitor (TFPI) and thrombomodulin (TM), suggesting that monocyte-derived EVs can themselves increase endothelial thrombogenicity. Although to a lesser extent, EVs isolated from platelets, granulocytes and endothelial cells were also able to exert high TF activity (Shustova et al., 2017). EC-derived EVs demonstrated the ability to induce TF expression and to increase the procoagulant activity of THP-1 monocytic cells (Sabatier et al., 2002). Circulating EVs additionally contribute to thrombosis through indirect mechanisms that are not associated with either TF or PtdSer surface expression. The use of Badimon perfusion chambers revealed EVs isolated from healthy blood volunteers, significantly enhanced platelet deposition on damaged arteries (Suades et al., 2012). Similarly, blood enriched with platelet-derived EVs, induced fibrin deposition on human atherosclerotic arteries and platelet adhesion to collagen-coated surfaces. Epinephrine/collagen closure time (critical in plaque formation) was significantly reduced with the addition of platelet-derived EVs, along with increased platelet aggregation in response to low doses of ADP and reduced clotting time. All together these results suggest that platelet-derived EVs, even under physiological conditions, can induce platelet activation and subsequent thrombus formation (Suades et al., 2012). EVs released from activated platelets may then, in turn, enhance the activation of non-activated circulating platelets and the endothelium (Barry et al., 1997).

In the last decade, a number of studies have demonstrated the role of EVs in angiogenesis and highlighted their therapeutic potential. Angiogenesis is a dynamic and heavily regulated process in which EC are in constant communication with their environment through multiple paracrine factors. EVs have the ability to induce angiogenesis, through the delivery of their specific cargo. As a relevant example, EVs mediated the transfer of miR-150 from monocytes to EC, and induced angiogenesis in these cells. Secreted miR-150 from THP-1-derived EVs entered EC and enhanced migration and tube formation. Moreover, they found that miR-150-containing EVs collected from the plasma of atherosclerotic patients enhanced angiogenesis *in vivo* (J. Li et al., 2013a). Besides monocyte-derived EVs, platelet-derived EVs also contributed to angiogenesis by way of cellular activation. This role was first described in 2004. Platelet-derived EVs isolated from healthy donors promoted proliferation, migration and tube formation of HUVEC through the cooperative effect of VEGF, FGF-2 and lipid components such as sphingosine-1-phosphate (Kim et al., 2004). Downstream signaling mechanisms involved in these effects were; the pertussis toxin-sensitive G protein and the PI3 kinase pathway. The proangiogenic activity of platelet-derived EVs was further supported by a variety of other studies. Platelet-derived EVs from Crohn’s Disease patients increased angiogenesis in a VEGF, FGF-2 dependent manner^122^. Platelet-derived EVs were also shown to be proangiogenic in an *in vivo* rat aortic ring model, due to the transfer of growth factors, such as VEGF, FGF-2 and PDGF; was dependent on PI3 kinase, src kinase, and ERK signaling events. Additionally, in a model of chronic myocardial ischemia in rats, platelet-derived EVs increased the formation of functional capillaries after injection into the myocardium (Brill et al., 2005). Platelet-derived EVs enhanced tumor progression and angiogenesis in a mouse lung cancer model, through the upregulation of factors associated with tumor vascularisation, such as IL-8, VEGF, and HGF (hepatocyte growth factor) (Janowska-Wieczorek et al., 2005). Recently, Sun et al., showed these EVs were able to promote HUVEC capillary-like network formation and migration in a dose-dependent manner. Moreover, levels of metalloprotease expression and activity; mostly MMP-2 and MMP-9, were upregulated in HUVEC. These pro-angiogenic and pro-migratory effects were abolished with the inhibition of MMPs (Sun et al., 2017). Angiogenesis can also be regulated by lymphocyte-derived EVs. These EVs strongly suppressed aortic ring microvessel sprouting and corneal neovascularisation. Moreover, they considerably diminished HUVEC survival and proliferation in a concentration-dependent manner *in vitro*, by augmenting ROS generation *via* NOX and interfering with the VEGF signaling pathway (Yang et al., 2008). Additionally, neutrophil-derived EVs play an active role in maintaining vessel integrity. Deposition of EVs during neutrophil extravasation was essential for maintaining endothelial barrier function during vascular inflammation (Lim et al., 2013). On the other hand, a recent study highlighted the involvement of neutrophil-derived EVs in atherogenesis, contributing to the plaque formation and progression by releasing miRNAs (Gomez et al., 2015).

## Extracellular Vesicles in Vascular Inflammation

It is not surprising that the role of EVs in vascular inflammation and in mediating vascular disease has been extensively investigated, with the most studied being platelet-derived EVs. Platelet-derived EVs carry similar, but not identical surface repertoire compared to their parent cells and can exert immunomodulatory effects on distant target cells (Varon and Shai, 2015). Several components such as growth factors (platelet-derived growth factor, TGF-β), enzymes (12-lipoxygenase, thromboxane synthase), cytokines e.g. IL-1, transcription factors and even functional mitochondria are found in platelet-derived EVs and can be efficiently internalised by other cells such as EC, macrophages, and neutrophils (Pierce, 1989; Brown and McIntyre, 2011; Laffont et al., 2013; Boudreau et al., 2014; Duchez et al., 2015; Laffont et al., 2016). Different mechanisms of internalisation are described for different cell types. For example, EC recognize PtdSer by receptors such as developmental endothelial locus-1 (Del-1) and the interaction of the receptor tyrosine kinase Axl with its ligand Gas6 found on the surface of EVs (Dasgupta et al., 2009; Happonen et al., 2016). The uptake by neutrophils seems reliant on 12-lipoxygenase activity present within EVs (Duchez et al., 2015). 12-lipoxygenase ablation prevents the internalisation of platelet-derived EVs within the arthritic joint and consistently decreases inflammation. Recently, another study showed the immunomodulatory role of platelet-derived EVs on CD4^+^ T cells during atherosclerosis (Sadallah et al., 2014). Moreover, platelet-derived EVs also harbor growth factors such as VEGF, FGF-2 and lipid factors, suggesting these vesicles may regulate angiogenesis (Brill et al., 2005). As previously mentioned, platelet-derived EVs have the interesting ability of enhancing cellular migration, proliferation, survival and tube formation in HUVEC, whilst concurrently reducing apoptosis (Kim et al., 2004). Platelet-derived EVs also increased the pro-angiogenic MMP-2 and MMP-9 in EC both *in vitro* and *in vivo* (Kim et al., 2004). Beyond their usefulness as possible markers of platelet activation, platelet-derived EVs have exhibited a plethora of properties that can be related to atherothrombotic disease, such as enhancing platelet and fibrin deposition on atherosclerotic arterial wall, promoting platelet adhesion, enhancing the recruitment of leukocytes and thrombus formation (Suades et al., 2012). Other important subsets of EVs have also been described as promoters of vascular inflammation. Neutrophil-derived EVs are implicated as triggers of atherosclerosis through the delivery of miR-155 to atheroprone endothelium and enhancing NF-κB expression (Gomez et al., 2018).

Endothelial-derived EVs were recently reviewed as a causative agent in vascular pathologies. They carry many proteins derived from their surface such as adhesion molecules: VE-cadherin, platelet endothelial cell adhesion molecule 1, ICAM-1, E-selectin, α-integrin, growth factors; endoglin, CD146, VEGF receptor, and hemostatic molecules; vWF, TF, TFPI, tissue plasminogen activator, plasminogen activator inhibitor 1, endothelial protein C receptor (EPCR). They also contain active components such as, endothelial NO synthase and urokinase type plasminogen activator (Dignat-George and Boulanger, 2011). The circulating levels of endothelial-derived EVs are thought to represent a balance between cell stimulation, proliferation, apoptosis and necroptosis. A number of different pro-inflammatory or coagulation stimuli, can increase their presence such as; TNF-α, LPS, ROS, plasminogen activator inhibitor, thrombin, camptothecin, C-reactive protein, uremic toxins, and estrogens (Brodsky et al., 2002; Šimák et al., 2002; Faure et al., 2006; Szotowski et al., 2007). Recently, endogenous NO and oxidised lipids (Nomura et al., 2004), were shown to enhance endothelial-derived EVs release coinciding with increased mechanical stress (especially shear stress), which could be an initiating event in endothelial dysfunction. Moreover, the expression of TF on endothelial-derived EVs could be induced (Kagawa et al., 1998; Combes et al., 1999; Šimák et al., 2002). With regards to vascular homeostasis, the expression of anionic phospholipids, especially PtdSer, contributes to their procoagulant role (Owens and Mackman, 2011). Endothelial-derived EVs can also bind monocytes, activate them and consequently induce TF expression (Combes et al., 1999). Conversely, a variety of studies have shown endothelial-derived EVs exhibit anticoagulant and vasculoprotective potential. They were able to deliver miR-126 or TXA_2_ to the vessel wall (Mostefai et al., 2008) and can express endothelial protein C receptor (EPCR) and activated protein C (APC); reported to have anticoagulant and cytoprotective effects by reducing apoptosis (Morel et al., 2009). Furthermore, endothelial-derived EVs can catalyse the generation of plasmin by plasminogen, which would support clot dissolution through increased fibrinolysis properties (Lacroix et al., 2007). In line with these divergent roles for endothelial-derived EVs, angiogenic properties have also been associated with endothelial-derived EVs, in this case concerning its impairment. LDLR^-/-^ mice on high fat diet were injected with both pathological and physiological concentration of endothelial-derived EVs, which reduced the activity of nitric oxide synthase, NO release, and angiogenesis (Ou et al., 2011). This inhibitory effect of EVs on vessel formation was also shown in diabetic patients with coronary artery disease (Tsimerman et al., 2011). Nevertheless, endothelial-derived EVs from HBMEC were found to promote angiogenesis at low concentration, through plasmin generation, whereas higher concentrations had the opposite effect (Lacroix et al., 2007). Perhaps the differing purported effects for endothelial-derived EVs on vessel formation, could be dependent on the concentration in each of the different models.

## EVs IN Tissue Resolution and Repair

The inflammatory process develops along three sequential and overlapping phases: inflammation, resolution, and repair (Headland and Norling, 2015). For decades, researchers had focused on uncovering mechanisms to block inflammation. It was believed that the cessation of inflammation and subsequent tissue repair was a passive process. In more recent years, many publications have uncovered this second phase is in fact orchestrated by a plethora of different mediators and immunological cells that contribute actively to this process (Sugimoto et al., 2016). Cellular-membrane derived counterparts have also been shown to be involved in processes that promote tissue resolution and repair.

It is well documented that immune cells are efficient modulators of other immune cells. Aside from direct cell contact, the use of messengers either released directly into solution or contained within EVs, deliver instructions to the recipient cell. The most prominent and established role of EVs is in triggering pro-inflammatory process however, it has been repeatedly demonstrated that EVs can have immunosuppressive and resolutive roles in various contexts. Admyre et al. revealed that during pregnancy, breast milk contains EVs positive for MHC classes I and II; CD63, CD81, and CD86. These MHC classes I and II positive EVs inhibited the production of anti-CD3-induced IL-2 and IFN-γ from allogeneic and autologous polymorphonuclear cells. These subsets of EVs also increased the numbers of Foxp3^+^CD4 ^+^CD25^+^ T regulatory cells (Treg) (Admyre et al., 2007). Reduced cytotoxicity of Natural Killer, CD8^+^, and γδ T cells was also demonstrated after incubation of these cells with placental exosomes bearing NKG2D ligands (Hedlund et al., 2009). Furthermore, EVs from amniotic fluid can modulate the cytokine production by monocytic cells (Bretz et al., 2013). This immunosuppressive role was also extensively associated with dendritic cells-derived EV. Dendritic cells-derived EVs were able to enhance CD4^+^CD25^+^ Treg activity (Yang et al., 2011). Dendritic cell-derived EVs overexpressing IL-10, blocked cell proliferation in a mixed lymphocyte reaction *in vitro* and delayed the onset of collagen-induced arthritis as well as suppressed arthritis progression in mice (Kim et al., 2005). Furthermore, the same group demonstrated that myeloid cell-derived EVs (MHCII^+^CD11b^+^EV) obtained from the blood of mice immunised with keyhole limpet hemocyanin antigen, reduced hypersensitivity upon a second challenge with the same antigen, suggesting that they might have an anti-allergic effect (Kim et al., 2007).

**Figure 3:**
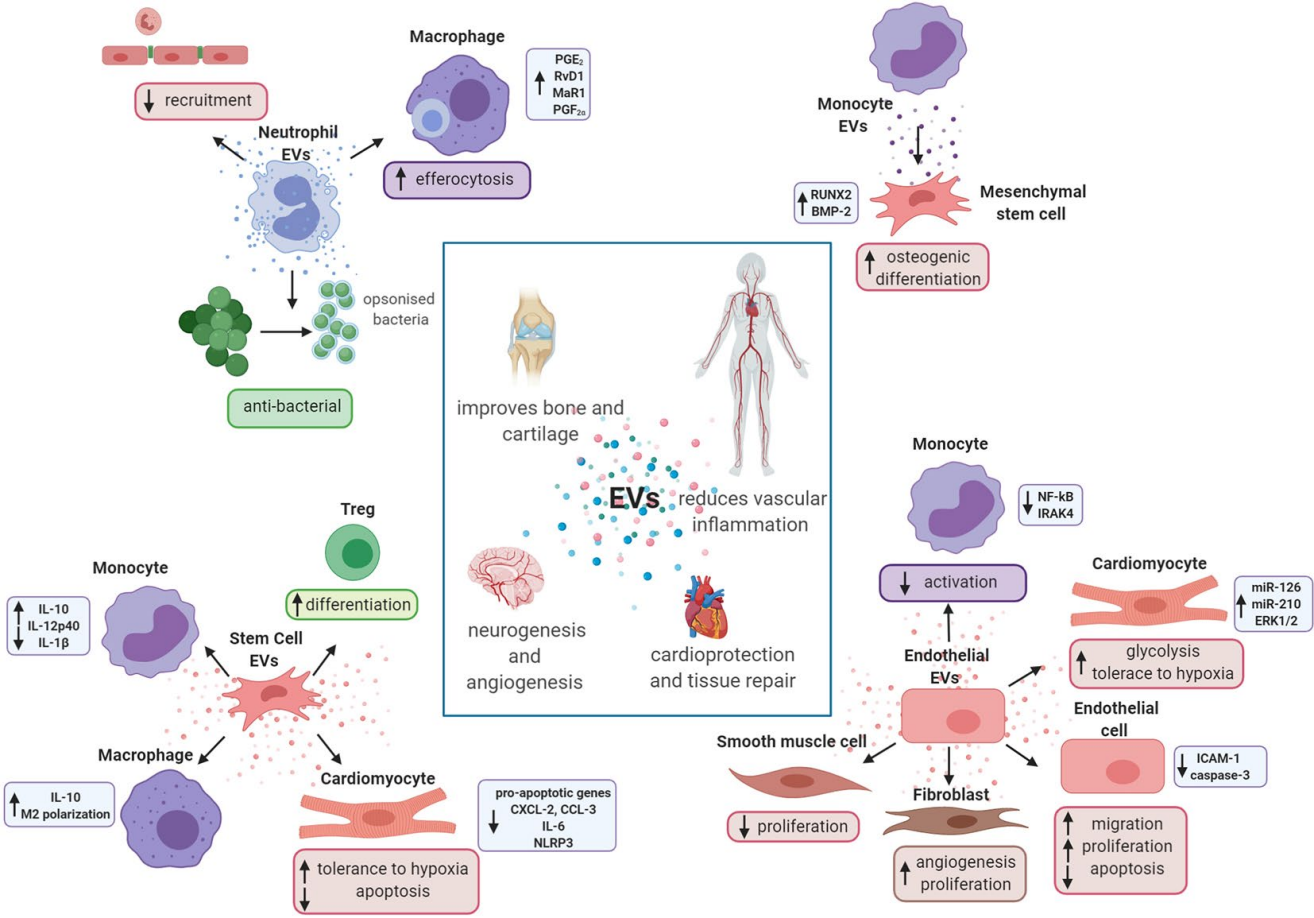
Extracellular vesicles promote tissue repair and regeneration. Schematic representation of the potential role of *in vitro*-generated extracellular vesicles (EVs) in tissue repair and resolution. Neutrophil EVs are able to promote cartilage protection and regeneration inducing pro-resolving mediators in macrophages and reducing the recruitment of cells to the endothelium. They also have antibacterial properties. Monocyte EVs are able to activate mesenchymal stem cells and enhance their osteogenic differentiation. Endothelial cell EVs promote angiogenesis and vascular repair, reducing activation of monocyte and smooth muscle cells. They are also able to increase glycolysis in cardiomyocytes and increase activation of endothelial cells and fibroblasts. Finally, stem cell EVs are shown to exert cardioprotective effects interacting with both leukocytes and cardiac cells.

Neutrophil-derived EVs released from activated neutrophils at the site of inflammation are shown to enhance antimicrobial activity *via* expression of opsonin CR1 receptor and of different antimicrobial proteins, such as MPO and human leukocyte elastase (Hess et al., 1998). These findings were further confirmed by Timar et al., where only EVs produced by stimulation of neutrophils with opsonised particles were capable of significantly reducing bacterial growth and their effect was independent of opsonisation and superoxide generation (**Figure 3**). Instead, these EVs seemed to form large aggregates with bacteria, a process dependent upon cytoskeletal reorganization, β2 integrin function, glucose metabolism and PI3-kinase activity of EVs. This effect was lost when neutrophils were stimulated with other pro-inflammatory stimuli (Lorincz et al., 2015). Equally, neutrophil-derived EVs were enriched in neutrophils granule proteins, many of which are known to be antibacterial (Timár et al., 2013).

The role of leukocyte-derived EVs in the resolution of vascular inflammation and cardiovascular diseases still lacks full investigation. This role has been previously reported by our group, both in the context of vascular inflammation and autoimmune diseases, such as rheumatoid arthritis. Dalli et al*.* reported significantly reduced neutrophil recruitment and adhesion to endothelial cell monolayers when neutrophils were pre-incubated with neutrophil-derived EVs. These vesicles contained the pro-resolving protein AnxA1 (Dalli et al., 2008). This self-limiting and anti-inflammatory mechanism has been reiterated in further studies, where neutrophil-derived EVs were shown to enhance the efferocytosis of apoptotic neutrophils and biosynthesis of specialised pro-resolving mediators (SPMs) in macrophages, including: PGE_2_, RvD1, MaR1, and PGF_2α_ (**Figure 3**). Interestingly, neutrophil-derived EVs do not contain mature SPMs (Dalli and Serhan, 2012), but contain SPM precursors esterified in EV membranes (Norling et al., 2011). Headland et al., demonstrated therapeutic roles of neutrophil-derived EVs in providing protection from cartilage damage in experimental arthritis *via* AnxA1. Elevated neutrophil-derived EVs present in the synovial fluid of rheumatoid arthritis patients were similarly enriched in AnxA1. Disruption of EV generation using the Tmem16f^-/-^ genetically modified mouse, resulted in impaired cartilage integrity during inflammatory arthritis (Headland et al., 2015). Recently, our group also demonstrated a direct role of neutrophil-derived EVs in counteracting the classical activation of macrophages, a role which was dependent upon PtdSer and AnxA1. These EVs also promoted the release of TGF-β (Rhys et al., 2018). Additionally, AnxA1^+^ neutrophil-derived EVs obtained from polymyalgia rheumatica patients (24 weeks post-steroid treatment) modulated neutrophil phenotype to suppress T-cell reactivity and proliferation (Nadkarni et al., 2019).

Furthermore, stimulation of human neutrophils, either in suspension or adherent to endothelial monolayers, led to the production of EVs with different proteomes, that differentially modulated endothelial genes (Dalli et al., 2013). Neutrophil-derived EVs have also been proposed to play a protective role in the prevention of vascular leakage during leukocyte extravasation, either by forming a physical barrier on the sub-endothelium or by inducing signaling events that regulate vascular barrier function (Lim et al., 2013). Interestingly, monocyte-derived EVs seem to be linked to tissue repair in bone regeneration. In 2013, Ekstrom et al., demonstrated that monocyte-derived Exos obtained from cells stimulated with LPS were able to induce osteogenic differentiation of mesenchymal stem cells (MSC), enhancing the expression of *RUNX2* and *BMP-2*; genes important in bone regeneration (**Figure 3**) (Ekström et al., 2013). Moreover, Exos derived from donor immature dendritic cells prolonged cardiac allograft survival by inhibiting T-cell activation. In this study, researchers found that the combination of immature dendritic cells and rapamycin notably increased the percentage of CD4^+^CD25^+^ T cells and up-regulated Foxp3 expression in recipient splenic T cells, but did not confirm whether these CD4^+^CD25^+^ cells were Tregs (Li et al., 2012b).

A number of studies have underlined the physiological role of endothelial-derived EVs acting as intercellular messengers, promoting endothelial regeneration and vascular protection *in vitro* and *in vivo*. Vascular resolution often appears to be promoted through the use of miRNAs. For example, a study reported the ability of EC-derived EVs to suppress monocyte activation, due to their anti-inflammatory miRNA content. In particular, miR-10a was identified as a possible candidate, shown to repress inflammatory signaling by targeting several components of the NF-κB pathway, including IRAK4 (Njock et al., 2015). Exos secreted from EC which overexpressed hypoxia-inducible factor 1 (HIF1), can be taken up by transplanted cardiac progenitor cells (CPCs) at the infarcted area (in a mouse model of MI) and increase miR-126 and miR-210 levels in CPCs (Ong et al., 2014). These mi-RNAs activated pro-survival kinases and induced a glycolytic switch in recipient CPCs, increasing the tolerance of these cells to hypoxic stress. Inhibiting both of these miRNAs blocked the protective effects of the Exos (Ong et al., 2014). This study highlights EV-miRNA trafficking as a possible mechanism of molecular cross-talk to promote resolution, one that could be utilised therapeutically. Moreover, Jansen et al., demonstrated the systemic treatment of mice with EC-derived EVs (isolated from starved human coronary artery EC), after electric denudation of the endothelium, accelerated re-endothelialisation. Whilst investigating the mechanism *in vitro,* they found that EC-derived EVs containing miR-126, promoted EC migration and proliferation through the active regulation of sprouty-related, EVH1 domain-containing protein 1 (SPRED1). This effect was abrogated both *in vitro* and *in vivo* when miR-126 was knocked down in EVs and when EVs were isolated from EC when glucose was reintroduced (Jansen et al., 2013). A similar effect of this specific miRNA delivered through EC-derived EVs was described also in VSMC (Jansen et al., 2017). In this second study, they demonstrated that systemic treatment of mice with EC-derived EVs after vascular injury reduced neointima formation *in vivo*. *In vitro*, this mechanism was found to be miR-126 transfer dependent from EVs, acting to reduce VSMC proliferation by binding to target protein LRP6; a co-receptor of the Wnt signaling pathway. Analysis of miR-126 expression in circulating EVs of 176 patients with coronary artery disease, revealed a reduced percutaneous coronary intervention rate in patients with high miR-126 expression levels, supporting a central role for EV-incorporated miR-126 in vascular remodeling. The same group also showed *in vitro* and *in vivo* anti-inflammatory actions of endothelial-derived EVs by promoting decreased expression of endothelial ICAM-1 through miRNA-222 transfer (Jansen et al., 2015). Furthermore, EC-derived EVs also appear to promote endothelial cell survival. Endothelial cells can actively remove caspase-3 *via* incorporation into EVs, thereby reducing intracellular levels of the pro-apoptotic caspase-3 (Hussein et al., 2005) (**Figure 3**). In line with this, endothelial and endothelial-regenerating cells were protected against apoptosis by AnxA1/PtdSer receptor-dependent EV uptake (Jansen et al., 2012). Similarly, human coronary artery EC exposed to EC-derived EVs, inhibited camptothecin-induced p38 activation. Moreover, endothelial-derived EVs containing endothelial protein C receptor and activated protein C were shown to promote cell survival by stimulation of cytoprotective effects (Pérez-Casal et al., 2009).

A recent publication has shown that EC have the ability to release cardioprotective Exos that may contribute to ischemic pre-conditioning. Cultured EC release EVs that were found to protect rat cardiomyocytes against subsequent exposure to hypoxia and reoxygenation (Davidson et al., 2018b). To address this mechanism of protection, the authors utilised an ERK1/2 inhibitor, as they previously demonstrated cardioprotection by plasma EVs was dependent on ERK1/2 signaling and activation of the TLR4 receptor pathway by HSP70 on the vesicle surface. However the exact mechanism by which HUVEC EVs activate ERK1/2 in cardiomyocytes remains unknown since these vesicles did not appear to contain HSP70 (Vicencio et al., 2015).

Endothelial progenitor cell-derived EVs are ascribed the ability to regulate fibroblast differentiation to an endothelial cell phenotype. Human endothelial progenitor cell-derived EVs were also able to enhance the proliferation and angiogenesis of cardiac fibroblasts (CF) *in vitro*. Furthermore, CF stimulated with these Exos displayed higher expression of the EC-specific markers, such as CD31 and VEGF2, whilst reducing the expression of proteins involved in fibrosis; alpha-smooth muscle actin, vimentin, collagen I, TFG-β, and TNF-α. In addition, CF stimulated with endothelial progenitor cell-derived EVs, resulted in reduced expression of astrocytic-high mobility group box-1. Despite these findings suggesting a major role of EC-derived EVs in vascular regeneration, many contradictory reports suggest they play a role in the promotion of vascular inflammation. These conflicting accounts are most likely due to EV protein and miR composition (Huber et al., 2002; Morel et al., 2008; Halkein et al., 2013; Yamamoto et al., 2015). Hosseinkhani et al., presented a good example of this dichotomous action. In their work, immunomodulatory content of EVs derived from unstimulated ECs and TNF-α induced ECs differently affected the inflammatory status of recipient THP-1 monocytic cells and HUVEC. ELISA assays revealed that TNF-α induced EVs contain a pro-inflammatory profile and chemotactic mediators, including ICAM-1, CCL-2, IL-6, IL-8, CXCL-10, CCL-5 and TNF-α compared to EVs from unstimulated cells. Furthermore, TNF-α induced EVs were able to selectively transfer functional inflammatory mediators that increased IL-6, IL-8 and ICAM-1 levels in recipient HUVEC and promoted the adhesion and migration of THP-1 cells. Taken together, these findings highlight EC-derived EVs can become enriched in a cocktail of inflammatory markers, chemokines and cytokines, able to establish a targeted cross-talk between EC and monocytes (Hosseinkhani et al., 2018).

In recent decades, multipotent MSC-derived EVs are widely reported as promoters of tissue repair in cardiac tissue and pathologies associated with vascular inflammation. For example, rat bone marrow MSC-derived Exos increased neurite remodeling, neurogenesis and angiogenesis in the ischemic boundary zone in an induced stroke model (Xin et al., 2013). These tissue repair effects were also seen in a rat model of traumatic brain injury, alongside reduced neurovascular inflammation (Zhang et al., 2017). Furthermore, in a mouse model of stroke, treatment with MSC-derived EVs presented with similar outcomes to MSC transplantation; moderating post-ischemia peripheral blood lymphopenia and skewing towards brain remodeling (Doeppner et al., 2015). Stem cell-derived EVs have also been described to have an immunosuppressive role in other vascular inflammatory syndromes. As a relevant example, Embryonic Stem Cells (ESC)-derived EVs were incubated with monocytes with a resultant increase in the release of the anti-inflammatory cytokine IL-10 and decreased production of pro-inflammatory cytokines IL-1β and IL-12p40 (**Figure 3**). It was also shown that co-incubation of MSC-derived EVs with THP-1 cells induced the differentiation of CD4^+^ CD25^+^ FoxP3^+^ Tregs (Zhang et al., 2014). Moreover, these EVs had an overall reducing effect on the severity of inflammation in a model of ischemia-induced myocardium tissue damage, contributing considerably to the protection of heart function. This was demonstrated with both local effects; lower neutrophil and macrophage infiltration into heart tissue, as well as systemic reduction in white blood cell counts (Arslan et al., 2013). Interestingly, the immunosuppressive action of these EVs could be modulated by preconditioning their parent cells. For example, when EVs were isolated from MSC stimulated with LPS, their progeny EVs were more effective at driving M2 polarisation of THP-1 cells and promoting closure of diabetic cutaneous wounds (Ti et al., 2015). Stem cell-derived EVs play a key role in reducing the extent of injury during myocardial ischemia reperfusion and in MI. In 2010 Lai et al., revealed purified Exos secreted by MSC could reduce myocardial ischemia/reperfusion injury using mouse models (Lai et al., 2010).

Various experimental studies have confirmed that engineered CD34^+^ stromal cells are also able to release Exos containing proangiogenic factors, conveyed to infarcted mouse myocardium decreased infarct size and improved long-term regeneration (MacKie et al., 2012; Marrachelli et al., 2013). Arslan et al., demonstrated that treatment with MSC-derived EVs in the post-MI period actually improved myocardial viability and decreased adverse ventricular remodeling by reducing oxidative stress and activating the PI3K/Akt pathway (Arslan et al., 2013). Another type of stem cell acquiring growing interest for their potential roles in cardiac tissue repair, are the cardiosphere-derived cells (CDCs). These cells show multi-lineage differentiation and are described in various preclinical models to improve cardiac function after delivery CDC-derived EVs to ischemic myocardium (De Couto et al., 2015; Tseliou et al., 2015). Curiously, the strong reduction in infarct size facilitated by CDC-derived EVs has correlated with EV-YF1. This RNA belongs to a family of small noncoding RNA called Y RNA which are active components of the Ro60 ribonucleoprotein particle involved in DNA replication. Interestingly, the transfer of EV-YF1 by EV from CDCs to macrophages resulted in an increased expression of the immunosuppressive cytokine IL-10 (Cambier et al., 2017). Although there is some mechanistic understanding, how EVs promote cardioprotection is yet to be fully revealed. It appears to be the result of a direct interaction with cells in the heart, rather than blood components. Numerous studies have underlined the regeneration of post-infarct myocardium could be mediated by miRNA and that this could be enhanced by EV delivery from both cardiac and noncardiac SC. Among the most studied are MSC-derived EVs containing miR-21 (Luther et al., 2018; Mayourian et al., 2018) and its different isoforms, which appear to be good candidates for cardiac tissue repair. MSC-derived EVs were very recently shown to exhibit cardioprotection by increasing the level of miR-21a-5p in recipient cardiac cells, thereby downregulating expression of the pro-apoptotic gene products PDCD4, PTEN, Peli1, and FasL in the myocardium (Luther et al., 2018). Another study highlighted that induced pluripotent SC-derived EV miR-21 and HIF-1α-regulated exosomal miR-210, can induce cytoprotective signals to cardiomyocytes in *in vitro* setting of myocardial ischemia/reperfusion (Wang et al., 2015b). In line with this, Feng Y et al., revealed that the upregulation of miR-22 in MSC exposed to ischemic preconditioning, led to the transfer of this miRNA into EVs. When these EVs were injected into infarcted mouse hearts, they improved the repair of tissue damage resulting from MI and the mechanism for this involved the downregulation of Mecp2 (methyl CpG binding protein 2) (Feng et al., 2014). Interestingly, the same profibrotic protein MEcp2 was found to be modulated by miR-132. This miRNA was first described in perivascular pericytes and was shown to be transferred in EC *via* an EV mediated mechanism. Furthermore, cardiac progenitor cell exosomal miR-451 was found to improve clinical outcomes when mouse hearts were injected with EVs after the induction of ischemia (Chen et al., 2013). Finally, EVs containing miR-223, the most highly expressed miRNA in bone marrow-derived MSCs, downregulated Sema3A and Stat3 and reduced levels of pro-inflammatory cytokines in a sepsis-triggered cardiac injury model. The reduction of cardiomyocyte death during sepsis and enhanced cardiac function were attributed to the action of miR-223, which was able to downregulate various inflammation related genes such as Mef2C, PKnox1, CXCL2, CCL3, IL-6, NLRP3, TANK, DR6, and IRF4 (Wang et al., 2015) (**Figure 3**). EVs released from other cell types, for example, injured cardiomyocytes have also been reported to contain some cardiac-specific miRNAs, particularly miR-1, miR-133a, and miR-208 and activate cardiac muscle cells and resident cardiac progenitor cells to initiate cardiac tissue repair (Chistiakov et al., 2012). Moreover, plasma exosomes isolated from human or rat blood by differential ultracentrifugation exhibit a cardioprotective effect, decreasing infarct size when administered intravenously to rats or primary cardiomyocytes. The same group established that the presence of HSP70 on the surface of EVs was able to activate the ERK1/2 pathway through stimulation of TLR4, and inhibition of these mechanisms led to reduced cardioprotection (Vicencio et al., 2015). This cardioprotective effect however, was completely lost when these EVs were isolated from the blood of rats or humans with type II diabetes, suggesting plasma EVs may continually exhibit, mild stimulatory effects on cardioprotective pathways in the heart during physiological conditions and not during vascular pathologies (Davidson et al., 2018a).

## Therapeutic Potential of EVs

The ubiquitous nature and endogenous properties of EVs poses them as novel therapeutic strategies in the treatment of diseases. EVs have the potential to generate efficacious effects in pathology with the added benefit of circumventing drug side-effects. For example, MSC-derived EVs were shown to enhance repair in damaged cardiac tissue (El Andaloussi et al., 2013), EVs were able to reduce infarct size and improve left ventricle ejection fraction following ischemia (Ciullo et al., 2019) and interestingly, EVs derived from DCs have been used as part of cancer immunotherapeutics (Zitvogel et al., 1998). It is not only the biological content of EVs that can be harnessed, but they can also act as scaffolds, providing the vehicle through which active compounds can be administered, masked by the membrane encapsulation of “self.” Human neutrophil-derived EVs, enriched with lipid mediators such as aspirin-triggered resolvin D1 or lipoxin A4 exhibited therapeutic properties including reduced neutrophil cell influx during *in vivo* peritonitis models and improved resolution of inflammation; demonstrated by accelerated keratinocyte healing (Norling et al., 2011).

## Concluding Remarks

Researchers have gleamed a wealth of information from studying EVs in health and disease, identifying their use as biomarkers of inflammatory diseases and exploring their repertoire of functions. We have highlighted here the yin and yang of EVs in the context of cardiovascular disease; promoting vascular inflammation yet also initiating resolution and repair mechanisms. We have iterated how the composition of EVs are dependent upon the parent cell status and active transfer of specific miRNA, receptors, enzymes, lipids, and proteins that yields functional vectors that influence the phenotype of recipient cells. Together, the breadth of information we have discussed in this article indicates the powerful nature of these tiny organelles and their utility to be manipulated for therapeutic purposes.

## Author Contributions

SO and SA-W contributed equally to writing this manuscript. All authors planned, edited, and reviewed the article prior to submission.

## Funding

SO is supported by EVOluTION that has received funding from the European Union’s Horizon 2020 research and innovation program under the Marie Sklodowska-Curie grant agreement No. 675111. SA-W and LN are supported by a Versus Arthritis PhD Studentship and Senior Research Fellowship (grants 21941 and 22235 respectively). LVN is also supported by Barts Charity (Project grant MGU0443). This work has been facilitated by the National Institute for Health Research Biomedical Research Centre at Barts Hospital NHS Trust.

## Conflict of Interest

The authors declare that the research was conducted in the absence of any commercial or financial relationships that could be construed as a potential conflict of interest.
